# Insulin Resistance Predicts Postoperative Cognitive Dysfunction in Elderly Gastrointestinal Patients

**DOI:** 10.3389/fnagi.2019.00197

**Published:** 2019-08-08

**Authors:** Xi He, Ge Long, Chengxuan Quan, Bin Zhang, Jia Chen, Wen Ouyang

**Affiliations:** Department of Anesthesia, The Third Xiangya Hospital of Central South University, Changsha, China

**Keywords:** insulin resistance, metabolic risk factors, postoperative cognitive dysfunction, elderly, tumor necrosis factor α

## Abstract

**Background:**

Members of the aging population who undergo surgery are at risk of postoperative cognitive dysfunction (POCD). Exploring an effective and reliable early predictor of POCD is essential to the identification of high-risk patients and to making prospective decisions. The purpose of this study was to examine whether preoperative insulin resistance is an independent predictor of POCD.

**Methods:**

A total of 124 patients aged 60 years and older and who were scheduled for gastrointestinal surgery were enrolled in a prospective observational clinical study. All participants completed a battery of neuropsychological tests before surgery and 7 days later. POCD was defined as a decline of at least 1.5 SD on two or more of neuropsychological tests. Plasma concentration of the tumor necrosis factor α (TNF-α), C-reactive protein (CRP), and S-100β protein were measured. The status of insulin resistance was assessed by Homeostasis Model Assessment–Insulin Resistance (HOMA-IR). The relationship between HOMA-IR and POCD was assessed by Multivariable logistic regression models and the receiver operating characteristic (ROC) curve.

**Results:**

Fifty one patients (41.1%) were diagnosed with POCD at 7 days after surgery. Preoperative HOMA-IR values of the POCD group were significantly higher than the non-POCD group. Furthermore, CRP and TNF-α levels of the POCD group were significantly higher at each postoperative time point (*P* < 0.05). The preoperative HOMA-IR value was an independent predictor of POCD (adjusted OR 1.88, 95% CI, 1.18–2.99) even after adjust for confounding variables, and when dichotomized, individuals above the HOMA-IR threshold (HOMA-IR > 2.6) had a three-times higher risk of POCD (OR 3.26; 95% CI, 1.07–9.91) compared to individuals below the threshold. The areas under the ROC curve of HOMA-IR was 0.804 (95% CI, 0.725–0.883; *P* < 0.001). The optimal cut-off value was found to be 0.583, with a sensitivity of 84.3% and specificity of 74%. The HOMA-IR value was positively associated with the TNF-α concentration at baseline (*R*^2^ = 0.43, *P* < 0.01) and 1 day after surgery (*R*^2^ = 0.3861, *P* < 0.01).

**Conclusion:**

Preoperative insulin resistance is an effective predictor for the occurrence of POCD. Targeted prevention and treatment strategies of insulin resistance may be effective interventions of patients at risk for POCD.

## Introduction

Postoperative cognitive dysfunction (POCD) is a common neuropsychological complication after surgery, with influences on various aspects of cognitive functioning, such as learning, memory, attention, and executive function, particularly in the elderly. Currently, the evaluation of POCD is based on a battery of neuropsychological test differences between pre- and postoperative performance and is often delayed. POCD has been confirmed to be associated with multiple poor outcomes, including increased surgical complications, prolonged hospitalization, impaired quality of life, increased risk of disability, and mortality (Lewis et al., 2004; Monk et al., 2008). Furthermore, POCD may accelerate the onset and progression of Alzheimer’s disease (Chen et al., 2014). However, to date, the underlying pathophysiology of POCD remains abstruse, being recognized as a result of interaction of multiple factors Thus, exploring the effective and reliable early predictor of POCD is essential to identifying high-risk patients and making prospective decisions.

Recently, there has been an increase in awareness on the contribution of metabolic risk factors such as obesity, diabetes, dyslipidemia to the occurrence of POCD (Feinkohl et al., 2016, 2017a,b, 2018). Indeed, Diabetes and obesity can affect the structure and function of the brain and show a much higher risk of cognitive impairment and even dementia (Bischof and Park, 2015; Schimming et al., 2017), especially in the elderly. Insulin resistance (IR) is not only at the core of obesity, diabetes, dyslipidemia, and metabolic syndrome (Sesti, 2006), but also represents a shared hallmark characteristic of neuropathological processes underlying cognition aging and Alzheimer’s disease (AD) (Frisardi et al., 2010; Heni et al., 2014; Akintola and van Heemst, 2015; Kullmann et al., 2016). There are numerous studies drawing a strong association between insulin resistance and poorer cognitive performance (Ekblad et al., 2017; Neergaard et al., 2017). In the central nervous system, insulin plays an important role in learning and formation of memory. Moreover, insulin resistance facilitates brain inflammatory responses and influences AD progression by increasing β-amyloid and reducing synaptic plasticity (Craft, 2005). Therefore, it may be that insulin resistance is a condition affecting both peripheral and central insulin signaling pathways, and insulin resistance constitutes a potential link between metabolic and cognitive dysfunction (Cholerton et al., 2013; Verdile et al., 2015). Regrettably, previous data only indicate that patients with metabolic risk factors may experience an increased risk of POCD; the influence of insulin resistance on POCD has not been proven in great detail. Therefore, exploring the relationship between insulin resistance and POCD is urgently needed through further research, especially in view of the modifiable nature that leaves room for risk reduction in high-risk individuals. These urged us to conduct the current research.

Thus, we aimed to evaluate the prospective association between insulin resistance and the subsequent risk of POCD, with a focus on whether insulin resistance is an independent predictor of POCD.

## Materials and Methods

### Participants and Study Design

This was a prospective observational clinical trial. All procedures of study were approved by the ethical committee of the Third Xiangya Hospital of Central South University, China (K18178). The study was registered on the Chinese Clinical Trial Registry (ChiCTR1800019768). All study participants provided written informed consent before enrollment.

Eligible patients were older than 60 years, classified under American Society of Anesthesiologists physical status I–III, and scheduled for elective gastrointestinal surgery lasting at least 2 h under general anesthesia. Additional inclusion criteria were that the participants must have the ability to communicate with the interviewer and complete a battery of neuropsychological testing. Exclusion criteria were any patients who had a preexisting psychiatric or neurological disease (e.g., intracranial tumors), had undergone surgery within the past 12 months, and registered a baseline Mini-Mental State Examination (MMSE) score < 23.

### Clinical Measurements

On admission, all patients who were accepted provided detailed history records such as self-reports of a diagnosis of hypertension, diabetes mellitus, heart disease, and previous stroke, a detailed list of medication and were administered a clinical examination. Clinical examination includes the measurement of weight, height, waist circumference and blood pressure, using an average of 3 seated blood pressure measurements.

The metabolic syndrome was defined using a modified version of the definition recommended by the American Heart Association (Alberti et al., 2009). The presence of any 3 out of 5 of the following risk factors would qualify a person for the metabolic syndrome: (1) elevated waist circumference (man ≥ 85 cm, woman ≥80 cm), (2) elevated triglycerides ≥1.7 mmol/L or use of medication for dyslipidemia, (3) reduced HDL-C (males <1.0 mmol/L, females <1.3 mmol/L), (4) elevated blood pressure (systolic pressure above 130 mmHg or/and diastolic pressure higher than 85 mmHg or using an antihypertensive drug treatment), and (5) elevated fasting glucose (≥100 mg/dL or ≥5.56 mmol/L) or drug treatment of elevated glucose.

The homeostasis model assessment index (HOMA-IR) was used to assess the degree of insulin resistance (Ekblad et al., 2017). The HOMA-IR index was calculated using the following formula: Fasting Insulin × Fasting Glucose/22.5. A baseline HOMA-IR index greater than 2.6 was predefined arbitrarily as insulin resistance (Nagpal et al., 2016).

### Plasma Biomarker Measurement

After admission, venous blood was obtained fasting in the morning for each patient and analyzed for biochemical parameters (triglycerides, HDL-C, fasting glucose and fasting Insulin levels) using standard laboratory techniques which is already a clinical routine in our hospital. At baseline and on postoperative days (PODs) 1, 3, and 7, blood samples were collected, processed by centrifugation, then were frozen at −70°C until assayed. Neuronal injury S100β protein and 2 biomarkers of inflammation CRP and TNF-α were measured in the central laboratory of our institution, using enzyme linked immunosorbent assay (ELISA) or turbidimetric inhibition immune assay.

### Neuropsychological Test

Subjects were first screened with SDS, MMSE to exclude subjects with serious depression or cognitive impairment. Those enrolled participants completed a battery of neuropsychological tests conducted by a trained interviewer before surgery and 7 days after surgery. This cognitive test battery consisted of Verbal Learning and fluency Test, Visuospatial Memory and Delayed Recall Test, Benton Judgment of Line Orientation, Trail Making Test Parts A and B, Digit Span and Digit Symbol Substitution Test, which primarily focus on memory, attention, and executive function. Thedetailed content of these cognitive tests have been described previously (Lin et al., 2014).

A postoperative neuropsychological disorder in a test has been defined as a deterioration of one standard deviation (SD) compared to the preoperative test results (namely “the 1 SD criterion”) (Rasmussen et al., 2001). The standard deviation of each test is calculated from the corresponding preoperative assessments of all patients. For individuals, we subtracted the preoperative from the postoperative score and divided the results by the corresponding SD to obtain a value called Z score for each individual test, these Z scores were then used for the assessment of POCD. Patients were diagnosed as POCD if their Z score was less than at least 1.5 SD on two or more of eleven neuropsychological tests (Rasmussen et al., 2001).

In addition to the above neuropsychological tests, the following tests were also administered: (1) MMSE was used to exclude pre-existing cognitive impairment, (2) the Self-rating Depression scale (SDS) and the Self-rating Anxiety scale (SAS) were used to assess anxiety and depression at baseline, (3) the Visual analog scales 0–10 were used to assess postoperative pain and the presence of mood disorder.

### Anesthesia and Surgery

During the entire perioperative period, all clinical management followed recognized clinical practice. No premedication was administered before surgery. All patients received general anesthesia. To ensure that all patients were under similar levels of anesthesia, the depth of anesthesia was monitored by the bispectral index, which is required to be maintained between 40 and 60. Except for this requirement, anesthetic agents and other aspects of management (blood pressure targets, use of vasoactive drugs) were at the attending anesthesiologist’s discretion. Generally, midazolam, sufentanil, neuromuscular blockade, and propofol were used for anesthesia induction, followed by volatile (sevoflurane) or/and intravenous (propofol) anesthesia. Endotracheal intubation was the most common form of airway maintenance. Postoperative pain therapy was most commonly treated with patient controlled analgesia device using sufentanil in the initial period. The surgery was carried out by skilled surgeons in a routine laparoscopic excision of gastrointestinal cancer. All aspects of the patient’s condition were documented in case report forms.

### Statistical Analyses

Descriptive statistics of variables were examined in patients with and without POCD. Quantitative data were expressed as the mean ± standard deviation. Categorical data were expressed as the number and percentage. Statistical differences between two groups were investigated using *t*-test or Wilcoxon rank sum test, as appropriate for continuous data. The chi-square test or Fisher exact test was used for categorical data. Univariate logistic regression models were used; those variables which have statistical significance (*p* < 0.05) were then analyzed with the multivariate model. Multivariable logistic regression models were fitted to adjust for the odds ratio (OR) values and 95% confidence intervals (CI) of the independent predictors of POCD. The odds ratio estimates were adjusted for confounders age and postoperative pneumonia, and were then additionally mutually adjusted for all other metabolic components. The linear regression analysis was used to illustrate the relationship of HOMA-IR, CRP, and TNF-*upalpha*. Receiver operating characteristic (ROC) curves were configured to determine the best threshold of HOMA-IR for optimal predictive sensitivities and specificities of POCD. SPSS 22.0 was used for data analysis, where values of *p* < 0.05 were considered statistically significant. Graphics were created with GraphPad Prism Software version 8.0 for Windows.

## Results

### Enrolled Patients and Clinical Characteristics

**FIGURE 1:**
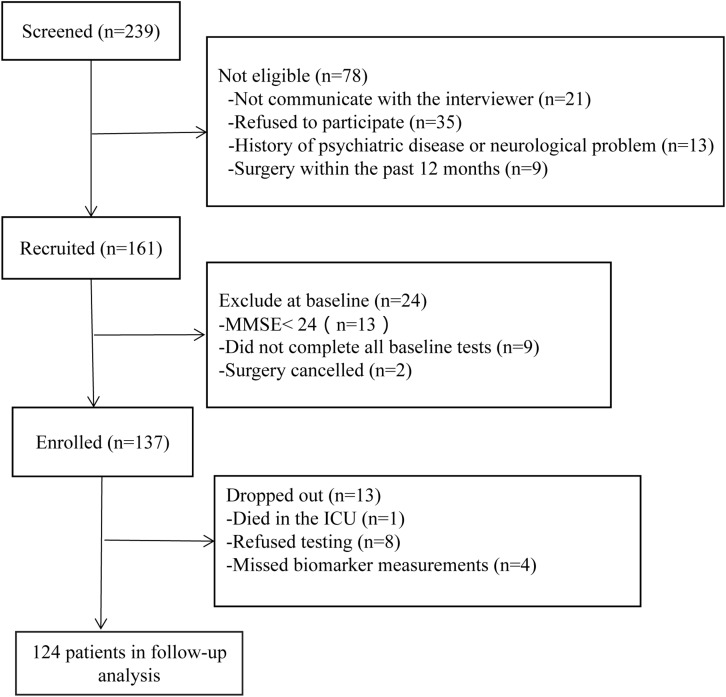
Enrollment and follow-up of study participants.

A total of 124 patients were included. The trial details are shown in Figure 1. The demographics and clinical characteristics of the participants are listed in Table 1. Fifty-one patients (41.1%) were diagnosed with POCD at 7 days after surgery. Compared to non-POCD, participants with POCD exhibited several abnormalities including elevated waist circumference (*p* = 0.002), elevated triglycerides (*p* = 0.083), and reported a higher incidence of pneumonia after surgery, but no surgery-related complications. Insulin and glucose level were marginally higher in the POCD group (*p* < 0.001). Furthermore, the prevalence of metabolic syndrome in patients with POCD (50.9%) was more than two times the prevalence in the non-POCD group (24.7%). Other cardiovascular risk factors such as hypertension, dyslipidemia, the level of physical activity, history of smoking, and alcoholism, were not statistically significant. Situations during perioperative period, such as ASA grade and the duration of time and blood loss, were observed with no significant difference (see Table 1).

**TABLE 1:** The demographic and clinical characteristics of the study participants.

	**ALL (*n* = 124)**	**POCD (*n* = 51)**	**Non-POCD (*n* = 73)**	***P*-value**
**Demographics**				
Age (y)	69.8 ± 5.4	71.1 ± 5.2	68.8 ± 5.3	0.027^∗^
Female, n (%)	60 (48.4%)	28 (54.9%)	32 (43.8%)	0.225
Height (cm)	160.7 ± 7.5	160 ± 8.1	161 ± 7	0.6
Weight (kg)	58.8 ± 10.1	60.9 ± 9.8	57.4 ± 10.2	0.06
Education (y)	6.7 ± 2.8	6.2 ± 3	7.1 ± 2.6	0.068
Smoking, n (%)	52 (41.9%)	22 (43.1%)	30 (41.0%)	0.821
Alcohol, n (%)	41 (31.1%)	18 (35.3%)	23 (32.4%)	0.186
**Physical activity**				
Inactivity	57 (45.9%)	25 (49%)	32 (43.8%)	0.681
1–2 times/week	25 (20.2%)	11 (21.6%)	14 (19.2%)	
≥3 times/week	42 (33.9%)	15 (29.4%)	27 (37%)	
**Metabolic and vascular factors**				
Type 2 diabetes mellitus	43 (34.7%)	23 (45.1%)	20 (27.4%)	0.042^∗^
Metabolic syndrome	44 (35.5%)	26 (50.9%)	18 (24.7%)	0.003^∗^
Self-reported cardiac disease	23 (18.5%)	11 (21.6%)	12 (16.4%)	0.47
Self-reported transient ischemic attack	11 (8.9%)	5 (9.8%)	6 (8.2%)	0.76
Waist circumference (cm)	83.7 ± 9.0	87.0 ± 9.7	81.4 ± 7.7	0.002^∗∗^
Systolic blood pressure (mmHg)	128.0 ± 17.0	129.3 ± 15.0	129 ± 15.4	0.918
Diastolic blood pressure (mmHg)	76.9 ± 10.2	78.0 ± 10.2	76.0 ± 10.1	0.277
Fasting plasma glucose (mmol/L)	6.0 ± 1.8	6.7 ± 2.3	5.6 ± 1.1	< 0.001^∗^
High density lipoprotein (mmol/L)	1.18 ± 0.3	1.1 ± 0.3	1.2 ± 0.3	0.019^∗^
Triglycerides (mmol/L)	1.6 ± 1.9	1.8 ± 1.1	1.5 ± 0.9	0.083^∗^
Insulin (mmol/L)	8.7 ± 4.0	11.3 ± 3.7	7.0 ± 3.2	< 0.001^∗^
HOMA-IR	2.5 ± 1.7	3.5 ± 1.7	1.9 ± 1.7	< 0.001^∗^
**Perioperative characteristics**				
Duration of surgery (min)	223.9 ± 76.8	221.5 ± 68.6	222.2 ± 75.8	0.96
Blood loss (ml)	217.7 ± 205.9	256.7 ± 242.4	185.8.0 ± 140.6	0.079
Hospital length of stay (d)	19.5 ± 6.0	20.4 ± 7.0	18.8 ± 5.2	0.113
Pneumonia	32 (25.8%)	19 (37.3%)	13 (16.9%)	0.015^∗^
Surgery-related complications	16 (12.9%)	7 (13.7%)	9 (12.3%)	0.884
Baseline SAS	24.3 ± 2.8	24.6 ± 2.7	24.1 ± 2.9	0.198
Baseline SDS	25 ± 3.2	24.7 ± 3	25.4 ± 3.5	0.313
Baseline MMSE	25.9 ± 2.5	25.4 ± 2.7	26.2 ± 2.3	0.085
VAS 1 day after surgery	4.40 ± 0.98	4.4 ± 0.99	4.3 ± 1.01	0.503

**Categorical variables are reported as absolute frequencies and percentages (round brackets), and using chi-square test. Continuous variables are reported as mean standard deviation, using the Student t-test or the Wilcoxon test appropriately. ^∗^p < 0.05; ^∗∗^p < 0.01; HDL, High density lipoprotein; POCD, Postoperative cognitive dysfunction; SDS, Self-rating Depression scale; SAS, Self-rating Anxiety scale; VAS, Visual analog scale for pain.**

### Neuropsychological Test Results

Preoperative SAS and SDS showed no difference between the two groups (Table 1).

The results of neuropsychological testing of participants at baseline and at 7 days after surgery were listed in Table 2. Participants with POCD exhibited worse performance mainly in Visuospatial Memory, Trail Making Test, and Digit Span Test.

**TABLE 2:** Neuropsychological test results at baseline and 7 days after surgery.

	**Baseline**			**7 days after surgery**		
		
	**POCD**	**Non-POCD**	***P-*value**	**POCD**	**Non-POCD**	***P-*value**
Hopkins verbal learning test-revised	13.4 ± 3.3	14.2 ± 2.5	0.182	10.6 ± 3.5	12.3 ± 2.7	0.003^∗∗^
Brief visuospatial memory test-revised	6.2 ± 2.2	6.6 ± 2.0	0.291	4.3 ± 2.4	5.4 ± 2.3	0.011^∗^
Trail-making test (Parts A and B)^#^	310.5 ± 81.1	287.6 ± 74.4	0.104	395.9 ± 113.1	336.7 ± 87.7	0.002^∗∗^
Benton judgment of line orientation	15.9 ± 2.8	15.5 ± 2.5	0.334	12.6 ± 3.0	14.0 ± 2.5	0.01^∗^
Digit span test	16.1 ± 2.9	15.8 ± 3.2	0.696	13.5 ± 3.4	15.1 ± 3.2	0.06
Symbol-digit modalities test	17.9 ± 4.9	19.1 ± 4.7	0.167	15.7 ± 5.4	17.7 ± 4.6	0.03^∗^
HVLT-R delayed recall test	3.7 ± 1.4	4.0 ± 1.2	0.99	2.7 ± 1.9	3.1 ± 1.3	0.152
HVLT-R discrimination index	22.3 ± 1.3	22.1 ± 1.8	0.549	21.3 ± 2.3	21.8 ± 1.5	0.183
BVMT-R delayed recall test	2.8 ± 1.1	3.0 ± 1.3	0.365	2.0 ± 1.8	2.2 ± 1.3	0.535
BVMT-R discrimination index	11.2 ± 4.1	11 ± 1.1	0.74	10.2 ± 4.3	10.6 ± 1.3	0.52
Verbal fluency test	38.8 ± 8.8	42.4 ± 8.7	0.034^∗^	35.2 ± 9.4	38.1 ± 8.5	0.105

*Continuous variables are reported as mean standard deviation, using the Student t-test or the Wilcoxon test appropriately. ^∗^ p < 0.05; ^∗∗^p < 0.01; ^#^In timed tasks, lower scores reflect better performance.*

### Primary Outcome: Relationship Between Metabolic Risk factors, IR and POCD

Table 3 presents the relations among metabolic risk factors, IR and incident POCD at follow-up. Insulin resistance was associated with an increased risk of POCD. The risk of POCD increased between 88% for every unit increase on the HOMA-IR index (OR 1.88, 95% CI, 1.18–2.99), and when dichotomized, individuals above the threshold of 2.6 had a three-times higher risk of POCD (OR 3.26; 95% CI, 1.07–9.91) compared to individuals below the HOMA-IR threshold. The relations among diabetes, metabolic syndrome, insulin resistance and POCD can be found in Supplementary Table S1. Furthermore, in our subgroup analyses by diabetics, we observed similar associations between HOMA-IR and the incidence of POCD in participants with diabetics (OR 2.38, 95% CI, 1.26–4.51) and participants who did not have diabetes (OR 2.48, 95% CI, 1.46–4.20) after adjustment for age and the other metabolic components (Table 4). At the same time, we also carried out a subgroup analysis based on obesity, dyslipidemia, hypertension, and metabolic syndrome. Subgroup analyses did not substantially change our findings (Table 4).

**TABLE 3:** Associations between metabolic risk factors, insulin resistance and POCD.

	**Crude OR**	**95% CI**	***P*-value**	**Multivariable adjusted OR**	**95% CI**	***P*-value**
**Metabolic characteristics**						
Fasting plasma glucose	1.638	1.235–2.174	0.01^∗^	0.984	0.679–1.424	0.93
Waist circumference (cm)	1.08	1.032–1.132	0.001^∗∗^	1.052	0.991–1.116	0.095
HDL	0.213	0.054–0.830	0.026^∗^	0.502	0.106–2.367	0.383
TG	1.379	0.95–2.003	0.091	NA		
**Cumulative sum of risk factors for Mets**						
5 component risk factors	3.385	1.310–8.752	0.012^∗^	3.13	1.21–8.14	0.019^∗^
Metabolic syndrome	3.437	1.599–7.389	0.002^∗∗^	0.991	0.332–2.961	0.987
Insulin resistance (HOMA-IR)						
Continuous	2.277	1.64–3.17	< 0.001^∗∗^	2.069	1.266–3.382	0.004^∗∗^
Dichotomized (HOMA-IR > 2.6)	6.89	3.05–15.58	< 0.001^∗∗^	3.26	1.07–9.91	0.037^∗^

**The model is adjusted for age, postoperative pneumonia, and the other metabolic risk factors. ^∗^p < 0.05; ^∗∗^p < 0.01; OR, Odds Ratio; CI, confidence interval.**

**TABLE 4:** Associations between Insulin Resistance and POCD in subgroup analysis based on metabolic members.

**Variable**	**All**	**POCD**	**Crude**	**95% CI**	***P*-value**	**Multivariable**	**95% CI**	***P*-value**
	**No**	**No**	**OR**			**Adjusted OR**		
**Diabetes**								
No	81	28	2.169	1.603–4.280	< 0.001^∗^	2.476	1.460–4.198	0.001^∗^
Yes	43	23	1.851	1.163–2.946	0.009^∗^	2.384	1.260–4.512	0.008^∗^
**Hypertension**								
No	70	30	2.016	1.292–3.147	0.002^∗^	1.8	1.007–3.010	0.025^∗^
Yes	54	21	2.817	1.611–4.924	< 0.001^∗^	3.461	1.605–7.462	0.002^∗^
**Central**								
**Obesity**								
No	74	24	1.995	1.344–2.962	0.001^∗^	2.43	1.466–4.028	0.001^∗^
Yes	50	27	2.122	1.252–3.596	0.005^∗^	2.007	1.015–3.968	0.045^∗^
**Dyslipidemia**								
No	72	27	2.459	1.538–3.932	< 0.001^∗^	2.699	1.486–4.904	0.001^∗^
Yes	52	24	1.939	1.245–3.020	0.003^∗^	2.074	1.153–3.730	0.015^∗^
**Metabolic**								
**Syndrome**								
No	80	25	1.868	1.271–2.744	0.001^∗^	1.863	1.208–2.875	0.005^∗^
Yes	40	26	2.494	1.326–4.690	0.005^∗^	3.579	1.521–8.424	0.003^∗^

**The model is adjusted for age and the other metabolic components ^∗^P < 0.05. OR, Odds Ratio; CI, confidence interval.**

**FIGURE 2:**
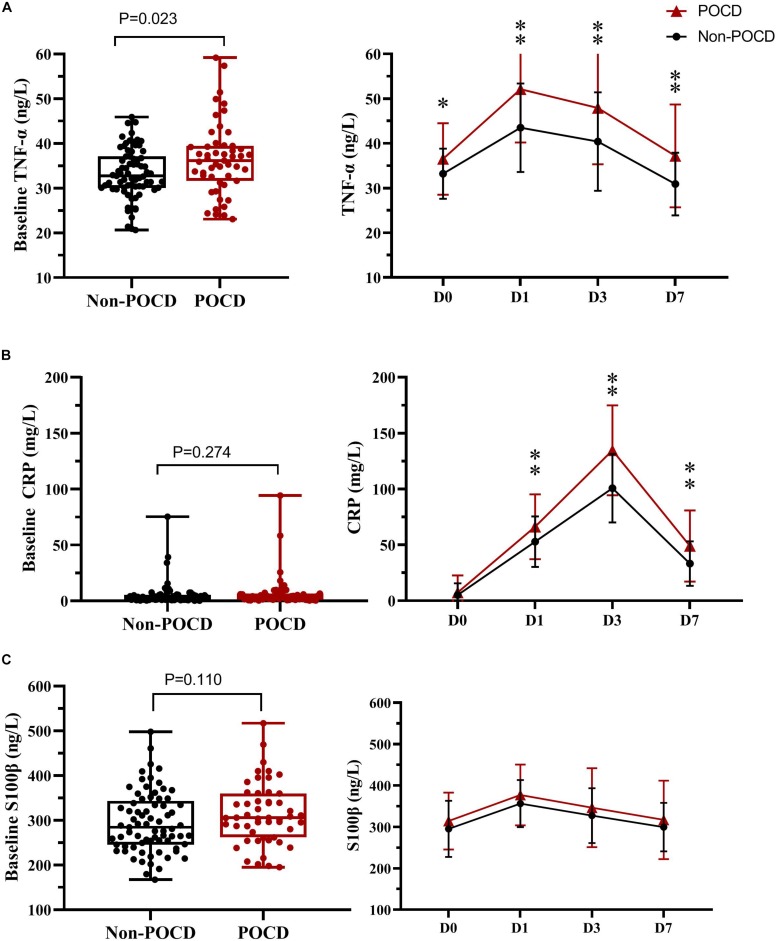
Receiver operating characteristic analysis of the preoperative HOMA-IR value. ROC, Receiver operating characteristic; HOMA-IR, Homeostasis Model Assessment–Insulin Resistance.

The ROC curves (Figure 2) shows that insulin resistance (assessed by the HOMA-IR) is the independent predictor of POCD. The areas under the curve of HOMA-IR was 0.804 (95% CI, 0.725–0.883; *P* < 0.001). The optimal cut-off value was found to be 0.583, with a sensitivity of 84.3% and specificity of 74%.

In addition, most metabolic risk factors showed a weak-to-moderate relationship with incident POCD after adjustment for age and postoperative pneumonia. However, after adjustment for all covariates and the other metabolic risk factors, associations were substantially meaningless. Only individuals with the worst metabolic condition, holding five risk factors for metabolic syndrome have a three-times larger odds (OR 3.13, 95% CI, 1.21–8.14) of developing POCD when compared to individuals with no metabolic risk factors. However, metabolic syndrome was not associated with an increased risk of incident POCD at follow-up.

### Plasma Concentration of CRP, TNF-α, and S-100β Results

**FIGURE 3:**
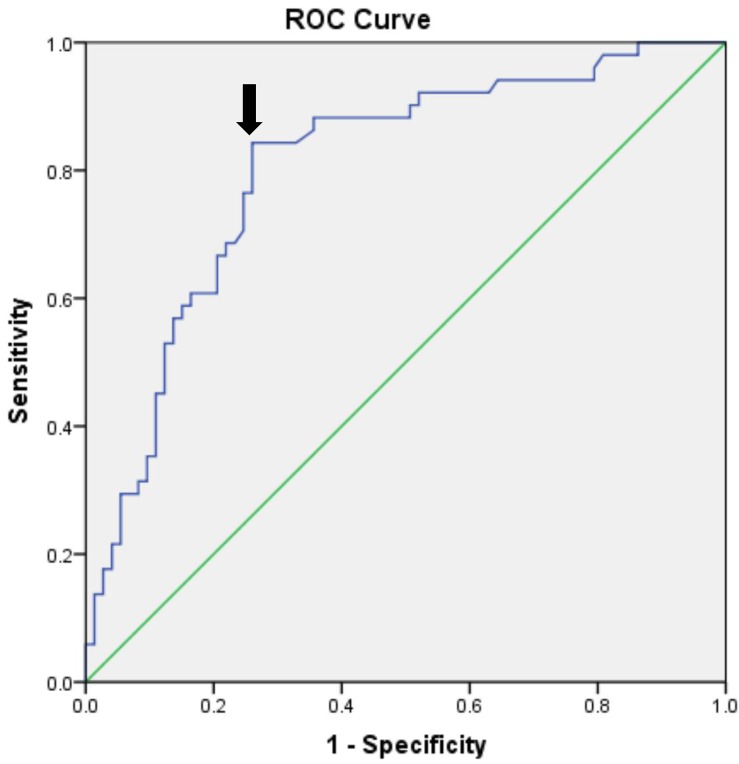
Plasma biomarker levels in two groups at different times. TNF-α **(A)**, CRP **(B)**, and S100β protein **(C)** were sampled at baseline and at 1, 3, and 7 days after surgery. ^∗^*p* < 0.05; ^∗∗^*p* < 0.01. POCD, postoperative cognitive dysfunction; Non-POCD, Non-postoperative cognitive dysfunction; TNF-α, Tumor necrosis factor α; CRP, C reactive protein; S100β, S100 calcium binding protein β. D0 at baseline; D1, 1 day after surgery; D3, 3 days after surgery; D7, 7 days after surgery.

**FIGURE 4:**
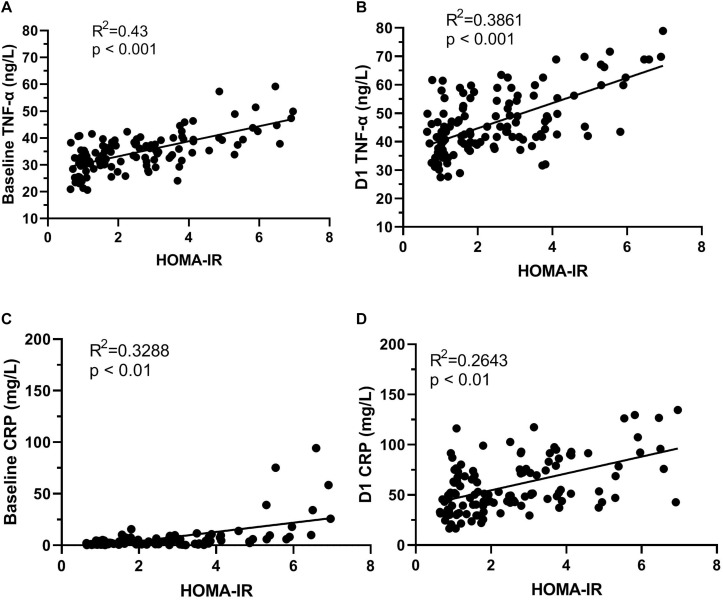
The correlation of HOMA-IR and systemic inflammation. HOMA-IR and TNF-α concentration at baseline **(A)**; HOMA-IR and TNF-α concentration D1 **(B)**. D1, HOMA-IR and CRP concentration at baseline **(C)**; HOMA-IR and CRP concentration D1 **(D)**. D1, 1 day after surgery.

The levels of plasma biomarkers of brain injury and systemic inflammation are listed in Table 5. No significant differences were observed in preoperative concentrations of CRP and S100β; only the TNF-α concentrations at baseline were higher in the POCD group. Compared to the non-POCD group (Table 5 and Figure 3), patients with POCD had higher systemic inflammation (high CRP and TNF-α) and neuronal injury (high S-100β) at each postoperative time point. Most importantly, as shown in Figure 4, according to the linear regression equation, the HOMA-IR value was positively associated with a TNF-α concentration at baseline (*R*^2^ = 0.43, *P* < 0.01) and 1 days after surgery (*R*^2^ = 0.3861, *P* < 0.01). Furthermore, the HOMA-IR value was also positively associated with CRP concentration at baseline (*R*^2^ = 0.3288, *P* < 0.01) and 1 day after surgery (*R*^2^ = 0.2641, *P* < 0.01), indicating a possible cross-talk between insulin resistance and perioperative inflammation.

**TABLE 5:** Plasma Biomarker Levels in Patients with and without Postoperative Cognitive Dysfunction (POCD).

	**Total**	**POCD**	**Non-POCD**	***P-*value**
**TNF-α (ng/L)**				
D0	34.6 ± 6.9	36.5 ± 8.0	33.2 ± 5.6	0.023^∗^
D1	47.0 ± 11.6	52.1 ± 11.9	43.5 ± 9.9	0.001^∗∗^
D3	43.5 ± 12.2	47.9 ± 12.6	40.4 ± 11	0.001^∗∗^
D7	33.5 ± 9.6	37.2 ± 11.5	30.9 ± 7	0.003^∗∗^
**CRP (ng/ml)**				
D0	6.2 ± 12.6	7.4 ± 15.3	5.3 ± 10.4	0.411
D1	58.9 ± 26.2	66.2 ± 29.1	53.7 ± 22.9	0.011^∗∗^
D3	114.7 ± 38.5	134.6 ± 40.2	100.7 ± 30.6	0.000^∗∗^
D7	39.8 ± 26.5	49.0 ± 31.8	33.28 ± 19.9	0.008^∗∗^
**S100β (ng/L)**				
D0	303.4 ± 68.5	314.2 ± 68.6	295.2 ± 67.8	0.110
D1	364.8.1 ± 64.6	377.0 ± 73.4	356.4 ± 56.7	0.088
D3	335.1 ± 79.6	346.4 ± 95.2	327.3 ± 66.2	0.309
D7	306.7 ± 75.7	329.0 ± 92.9	294.0 ± 54	0.436

**Continuous variables are reported as mean ± SD, using the Student t-test or the Wilcoxon test appropriately. ^∗^p < 0.05; ^∗∗^p < 0.01; TNF-α, Tumor necrosis factor α; CRP, C reactive protein; S100β, S100 calcium binding protein β. D0, before surgery; D1, 1 day after surgery; D3, 3 days after surgery; D7, 7 days after surgery.**

## Discussion

In this study, we found a high prevalence of preexisting insulin resistance in older patients undergoing gastrointestinal surgery, and most importantly, preexisting insulin resistance was associated with subsequent incident POCD. This association was independent of age, postoperative pneumonia, as well as all other metabolic derangement components. These findings suggest that preexisting insulin resistance associated with risk of POCD independently.

Insulin resistance is not only a shared hallmark characteristic of metabolic disease, but also a shared neuropathological process underlying cognition aging and AD.

Many studies have drawn a strong link between insulin resistance and cognitive decline (Akintola and van Heemst, 2015; Bischof and Park, 2015; Schimming et al., 2017), especially in the elderly. Indeed, peripheral hyperinsulinemia and insulin resistance can decrease insulin receptors expression at the blood-brain barrier, and reduce the insulin transport into the brain (Schwartz et al., 1990; Sartorius et al., 2015). These disturbances of insulin action can directly contribute to cognitive impairment and even AD. Currently, some researches described the potential association between AD and POCD (Arora et al., 2014; Evered et al., 2016). Postmortem studies of brain tissue on individuals with AD but not T2D have provided clear evidence showing that disturbance in insulin signaling, including decreased insulin levels and insulin sensitivity, as well as aberrant activation of the insulin receptor substrate (Biessels and Reagan, 2015), which also could be proved the relationship between insulin resistance and cognitive. In the present study, we find that the HOMA-IR value in individuals with POCD is higher than in those without POCD before operation, and individuals above the HOMA-IR threshold (HOMA-IR > 2.5) had three times larger odds of POCD. Although our study cannot draw causal conclusions, these results, combined with the previous pathological feature of AD, indicate the involvement of insulin resistance in POCD. Furthermore, evidence has accumulated that individuals with insulin resistance correspond to hippocampal atrophy, a sign of neurodegeneration (Ursache et al., 2012). Moreover, individuals with decreased peripheral insulin sensitivity have an impaired neuronal metabolism (Karczewska-Kupczewska et al., 2013) and brain glucose hypometabolism, evaluated by FDG-PET (Ohara et al., 2011; Thambisetty et al., 2013a,b). Therefore, the improvement of whole body insulin resistance could be a possible therapeutic option for neurodegeneration. Encouragingly, clinical research found that increasing CSF insulin by intranasal administration benefits the cognitive function in AD and mild cognitive impairment (MCI) patients (Craft et al., 2012; Claxton et al., 2013, 2015. In addition, animal experiments have shown that intranasal insulin administration prevent anesthesia-induced spatial learning and memory deficit in mice (Zhang et al., 2016), and this treatment in mice not only enhances object memory but also yields anxiolytic effects on behavior (Marks et al., 2009). All of these support insulin resistance as a key risk factor for the occurrence of POCD.

Currently, many animal studies have indicated that insulin and insulin resistance play a prominent role in amyloid beta metabolism and influence AD pathology (Cholerton et al., 2013; Verdile et al., 2015). However, the relationship between insulin resistance and amyloid load remains somewhat controversial in clinical research. In a longitudinal research, which included multiple OGTTs, PiB-PET data, as well as postmortem brain slices, amyloid accumulation was not confirmed to be relevant with either insulin resistance or glucose intolerance (Thambisetty et al., 2013b). Furthermore, AD-related regions with hypometabolism were observed; however, there were no increase in amyloid accumulation in T2D subjects (Roberts et al., 2014). This led us to draw a conclusion that there is no consistent pattern between amyloid accumulation and insulin resistance, which is also supported by research in patients with MCI or AD (Moran et al., 2015). Unfortunately, in our study, we did not evaluate amyloid accumulation in plasma or in CSF. Further studies with the evaluation of amyloid in blood or in CSF are necessary and useful to confirm (or not) literature data. In addition, this could represent a further investigation of the relationship between insulin resistance and POCD in terms of amyloid brain deposition.

Age has been shown to be an independent risk factor of POCD repeatedly (Monk et al., 2008). However, our study found no strong relation between age and POCD when the logistic regression model included HOMA-IR and age. In other words, if HOMA-IR was excluded from the logistic regression model, age was importantly associated with POCD, but inclusion of HOMA-IR weakened the relationship of age with POCD. This suggests that age could be acting as a substitute for insulin resistance status to a certain extent in analyses for risk of POCD. Previous research supports our results. Aging has been identified to be associated with insulin resistance, altered blood-brain barrier function for transport of insulin from the periphery to the brain decreased, and the direct effect on insulin receptor expression and activation; all of these might be responsible for insulin resistance in older individuals (Biessels and Reagan, 2015). Importantly, the elderly with insulin resistance are more susceptible to cognitive impairment and show an AD-like brain (Sheline and Raichle, 2013).

Many elderly individuals suffer from multiple metabolic diseases, such as diabetes, hypertension and abdominal obesity (Carracher et al., 2018; Bluher, 2019). These can directly result in the prevalence of insulin resistance which is probably over-represented in the surgical population and increases the risk of postoperative complications (Tzimas et al., 2015). Furthermore, the influence of metabolic derangement components on brain changes has been well proven and presents a higher risk of POCD (Feinkohl et al., 2016, 2017a,b, 2018). With metabolic syndrome considered to be a clustering of risk factors such as obesity, elevated blood glucose, elevated blood pressure, and dyslipidemia within a single individual, appear to confer an even greater risk for cognitive impairment than the sum of its individual components (Yaffe, 2007). The results of our study only support that individuals with a poor metabolic condition, holding five metabolic and vascular risk factors, have three-times larger odds of developing POCD than individuals with an ideal metabolic condition. However, the presence of a single metabolic member and metabolic syndrome itself does not provoke an increased risk of incident POCD at follow-up. All of these suggest that metabolic health is closely associated with brain health.

As noted in previous work, both clinical and preclinical studies indicate that inflammatory reactions could be responsible for incident POCD (Krenk et al., 2010; Skvarc et al., 2018). The results of our study support the potential role of increased systemic inflammation (high CRP and TNF-α) in the incident of POCD, but failed to show the relationship between elevated S100β and POCD. There are many possible mechanisms underlying this association; insulin resistance is supposed to play an important role. Indeed, peripheral hyperinsulinemia and insulin resistance facilitate brain inflammatory responses and influence AD pathology by increasing β-amyloid (Craft, 2005). In turn, these inhibit insulin receptors by activating TNF-α (Ferreira et al., 2014). How may inflammation account for the deleterious effect of insulin resistance on cognitive function? Most likely in theory, insulin resistance is associated with an inflammatory response and in turn, either the insulin resistant or inflammation or both, contribute to cognitive decline (Grundy, 2003; Ridker et al., 2003). Our findings are consistent with these results. We observed that the HOMA-IR value is positively associated with TNF-α and CRP concentrations at baseline and 1 day after surgery, respectively. Recently, Feng et al. (2013) found that surgery results in exaggerated neuroinflammation and persistent cognitive decline in a Rat Model of the Metabolic Syndrome. Moreover, blocking TNF-α in mice can effectively improve insulin sensitivity and prevent surgery-induced cognitive dysfunction (Terrando et al., 2010; Ferreira et al., 2014). These animal experiments corroborate our results that the negative impact of insulin resistance on inflammatory reactions could be accelerated because of surgical trauma, and some of the increased risk of POCD associated with insulin resistance is modified by inflammation.

Our study has limitations. One limitation is the lack of data on the ratio of CSF to serum insulin level which is closely associated with brain insulin resistance, because the influence of insulin resistance on cognitive function is mainly on the brain insulin signaling pathways. In addition, we did not evaluate amyloid beta accumulation in plasma or in CSF. This could represent a further investigation of the relationship between insulin resistance and cognitive impairment in terms of amyloid brain deposition. Additional studies with more complete data are necessary. Investigations should include POCD at 3 months and activities of daily living (ADLs). All of the above may be in favor to determine the exact contribution of insulin resistance to POCD.

## Conclusion

In conclusion, insulin resistance is associated with an increased risk of POCD in the elderly. This observation of a link between insulin resistance and POCD suggests that insulin resistance is an effective predictor of incident POCD. Thus, consideration of insulin resistance status may help clinicians and patients to make prospective decisions. The findings warrant further direction of research, particularly with respect to the underlying mechanisms and possible treatment strategies of POCD.

## DATA AVAILABILITY

The data for this manuscript will be made available by the authors to qualified researchers upon reasonable request. Requests to access the data should be directed to the corresponding author.

## Ethics Statement

All participants provided a signed written informed consent before enrolment in the study. All procedures described in this study were carried out in accordance with the declaration of Chinese Clinical Trial Registry (ChiCTR1800019768).

## Author Contributions

WO and XH designed the clinical experiment. WO directed the research group in all aspects. XH was the main investigator in this research. CQ contributed to sample selection and provision. BZ and JC were responsible for data acquisition. XH, GL, and WO carried out the data analysis and interpretation, and drafted the manuscript. All authors revised the manuscript.

## Conflict of Interest Statement

The authors declare that the research was conducted in the absence of any commercial or financial relationships that could be construed as a potential conflict of interest.
