# Supplementary material for: Insulin Resistance Predicts Postoperative Cognitive Dysfunction in Elderly Gastrointestinal Patients
Source: Front Aging Neurosci. 2019 Aug 8;11:197. doi: 10.3389/fnagi.2019.00197 (PMC6694405; doi:10.3389/fnagi.2019.00197)
Supplement: Supplementary file 1 [file Table_1.doc]

Table S1:

| Associations between Diabetes, Insulin Resistance and POCD | | | | | | |
| --- | --- | --- | --- | --- | --- | --- |
| Variable | Crude OR | 95% C.I. | P value | Multivariable Adjusted OR | 95% C.I. | P value |
| Type 2 diabetes mellitus | 0.485 | 0.231-1.020 | 0.057 | 2.127 | 0.688-6.577 | 0.19 |
| Hypertension | 0.827 | 0.399-1.712 | 0.608 | 2.067 | 0.743-5.754 | 0.164 |
| Central obesity | 0.354 | 0.168-0.746 | 0.006* | 0.668 | 0.218-2.048 | 0.481 |
| Dyslipidemia | 0.542 | 0.261-1.126 | 0.542 | 1.065 | 0.398-2.851 | 0.9 |
| Metabolic Syndrome | 0.291 | 0.135-0.625 | 0.002* | 0.359 | 0.087-1.486 | 0.158 |
| Insulin Resistance (HOMA-IR) | 2.157 | 1.574-2.956 | <0.001* | 2.094 | 1.445-3.033 | <0.001* |
| The model is adjusted for age , postoperative pneumonia, and the other metabolic components. * P < 0.05.  OR, Odds Ratio; CI, confidence interval. | | | | | | |
